# Fatigue Life Prediction of 2024-T3 Clad Al Alloy Based on an Improved SWT Equation and Machine Learning

**DOI:** 10.3390/ma18020332

**Published:** 2025-01-13

**Authors:** Zhaoji Li, Weibing Dai, Haitao Yue, Chenguang Guo, Zijie Ji, Qiang Li, Jianzhuo Zhang

**Affiliations:** 1 School of Mechanical Engineering, Liaoning Technical University, Fuxin 123000, China; 2 China Coal Technology & Engineering Group Shanghai Research Institute, Shanghai 200000, China

**Keywords:** Al alloy, extreme gradient boosting, random forest, fatigue life, deep deterministic policy gradient

## Abstract

The multi-parameter and nonlinear characteristics of the Smith Watson Topper (SWT) equation present considerable challenges for predicting the fatigue life of 2024-T3 clad Al alloy. To overcome these challenges, a novel model integrating traditional fatigue analysis methods with machine learning algorithms is introduced. An improved SWT fatigue life prediction equation is developed by incorporating key factors such as the mean stress effect, stress concentration factor, and surface roughness coefficient. Extreme gradient boosting, Random Forest, and their derived models are used to construct the fatigue life prediction model. The L-BFGS algorithm was then integrated with the established machine learning model to solve for the multi-parameter of the improved SWT equation. Thus, an accurate modified SWT prediction equation for 2024-T3 clad Al alloy was obtained. To further optimize the solution, the deep deterministic policy gradient and deep reinforcement learning algorithms are introduced to dynamically optimize the nonlinear equation, achieving a more efficient and accurate solution. The improved SWT fatigue life prediction equation and its solution method proposed in this study provide new insights for fatigue life prediction of clad metallic materials.

## 1. Introduction

Al alloys are widely used in aerospace, automotive manufacturing, military equipment, and high-end structural engineering due to their excellent strength-to-weight ratio, good corrosion resistance, and superior machinability [1,2]. Particularly in applications requiring lightweight and high-strength materials, Al alloys have become an indispensable choice [3,4]. However, despite their many outstanding properties, Al alloys still face challenges related to fatigue damage in practical applications. Under repeated cyclic loading, Al alloys are prone to fatigue crack formation, which can lead to structural failure [5,6]. This not only affects the service life of components but also poses significant safety risks [7]. Fatigue life prediction, as a key research direction in materials science and engineering, is crucial for improving the reliability and safety of Al alloy structures [8]. Accurate fatigue life prediction can help engineers optimize material selection and structural design during the design phase, reduce the cost and time of experimental testing, and effectively prevent accidents caused by fatigue failure [9]. However, the fatigue behavior of Al alloys is influenced by various complex factors, including the material’s microstructure, surface condition, stress concentration, and environmental conditions [10]. Traditional fatigue prediction models often have limitations when dealing with these complex factors. Therefore, developing more precise and comprehensive methods for predicting the fatigue life of Al alloys has become a pressing scientific challenge.

Many researchers have proposed various fatigue life prediction models to improve accuracy and broaden their application. These classical models include the M-C (Manson Coffin) equation [11], the Morrow elastic stress correction model [12], the M-H (Manson Halford) stress correction model [13], and the SWT (Smith Watson Topper) model [14]. The M-C equation is widely used due to its simplicity and ease of application. However, this equation is based solely on experimental data with a stress ratio of *R* = −1 and does not account for the influence of mean stress on fatigue life. Research indicates that mean stress exerts a notable adverse impact on the fatigue behavior of materials [15,16]. To address this, Morrow proposed the elastic stress correction model to improve the M-C equation by considering the effect of mean stress. However, the Morrow model has a limited scope and provides low accuracy in predicting the fatigue life of materials like Al alloys [17]. To overcome this, Manson and Halford further improved the Morrow model, proposing the M-H stress correction model, which considers the effect of mean stress on the plastic portion and enhances the prediction capability of the Morrow model [13]. However, the M-H model still fails to consider the impact of mean stress on damage control parameters (variables that reflect material degradation), leading to lower accuracy for some materials [18]. Subsequently, Smith et al. [14] introduced the SWT model, which classifies material fracture modes into shear and tensile types. For tensile-type failure, the SWT model can better describe the role of stress and strain in fatigue. Although the SWT model shows good predictive performance for materials with tensile cracking as the primary fatigue failure mode, it neglects the sensitivity of different materials to mean stress, resulting in overly conservative predictions in some cases [19,20]. Despite providing an effective framework for evaluating fatigue behavior, traditional fatigue life prediction models still face significant uncertainty and errors when dealing with complex loading conditions, material characteristics, and environmental changes [21]. Moreover, traditional models often rely on a large amount of experimental data to determine parameters [22,23]. The experimental process is both cumbersome and time-consuming, and model accuracy may be reduced in practical applications due to variations in material heterogeneity, manufacturing processes, and other factors [24]. Therefore, traditional methods still have considerable room for improvement.

In recent years, machine learning, as a powerful data-driven method, has been widely applied in the field of metal fatigue life prediction [25,26]. Machine learning methods, especially ensemble learning algorithms such as extreme gradient boosting (XGBoost) and Random Forest (RF), can effectively extract nonlinear relationships from large amounts of experimental data and provide efficient predictions [27]. However, relying solely on machine learning methods for fatigue life prediction, although demonstrating high prediction accuracy in some aspects, also has certain limitations. First, machine learning models often lack sufficient physical meaning and interpretability [28]. Their internal processes are typically black-box, making it difficult to directly relate the prediction results to the material’s micro-mechanical behavior [29]. Second, machine learning methods often require a large amount of experimental data for training, and the quality and quantity of data directly affect the prediction accuracy of the model [30]. Therefore, although machine learning methods can provide good predictive performance under certain conditions, their application in real engineering problems still faces many challenges. Combining machine learning with physical models for fatigue life prediction is the key to improving the prediction accuracy.

This study develops a predictive model for estimating the fatigue life of 2024-T3 clad Al alloy by integrating conventional fatigue approaches with machine learning techniques. The improved SWT fatigue life prediction equation is established by incorporating the physical foundation of the traditional SWT model and considering key factors such as mean stress effect, stress concentration factor (*K_t_*), and surface roughness coefficient (*C_s_*). The 2024-T3 clad Al alloy prediction model is then developed using machine learning algorithms, including XGBoost, RF, and their derived models. The L-BFGS algorithm is then integrated with the established machine learning model to solve for the unknown parameters in the improved SWT equation, resulting in an accurate modified SWT prediction equation for 2024-T3 clad Al alloy. Additionally, a deep reinforcement learning algorithm (DDPG) is introduced to dynamically optimize the nonlinear equations in the improved SWT model, achieving an efficient solution to the complex nonlinear improved SWT equations. Experimental results show that the proposed fatigue life prediction model not only overcomes the limitations of traditional methods but also significantly improves prediction accuracy, providing a more reliable solution for engineering applications of Al alloy fatigue behavior.

## 2. Experiment

In this study, the selected experimental material was 2024-T3 clad aluminum alloy with a surface roughness of *Ra* = 0.8 µm. The material was supplied by an aircraft manufacturing company, and its plate dimensions were 915 mm × 380 mm × 1.6 mm. The chemical composition and mechanical properties are detailed in Table 1 and Table 2. These data are derived from our previous research findings [31].

**Figure 1:**
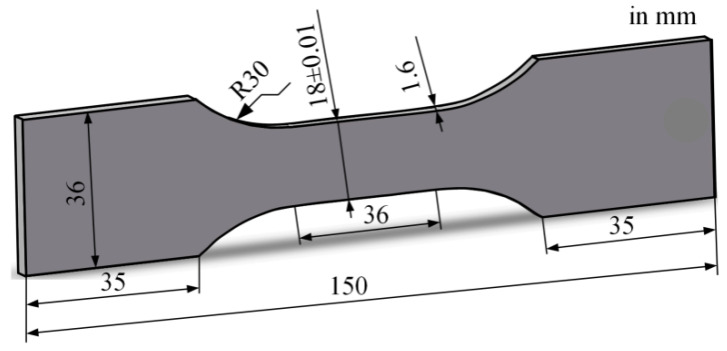
Geometry of the fatigue specimens.

During fatigue test specimen preparation, wire electrical discharge machining (WEDM) was applied to avoid deformation of the Al plates. The dimensions of the samples are presented in Figure 1. In addition, the wire-cut samples underwent ultrasonic cleaning with deionized water to mitigate surface corrosion caused by residual cutting fluid, followed by subsequent drying. After sectioning the workpieces, the wire-cut surfaces were carefully polished using sandpapers with grit sizes from 400 to 2000 to achieve smooth finishes. Subsequently, the specimens were further cleaned with an ultrasonic cleaner using alcohol and finally dried with a blower. The hardness of the specimen is 38 HRB.

**Figure 2:**
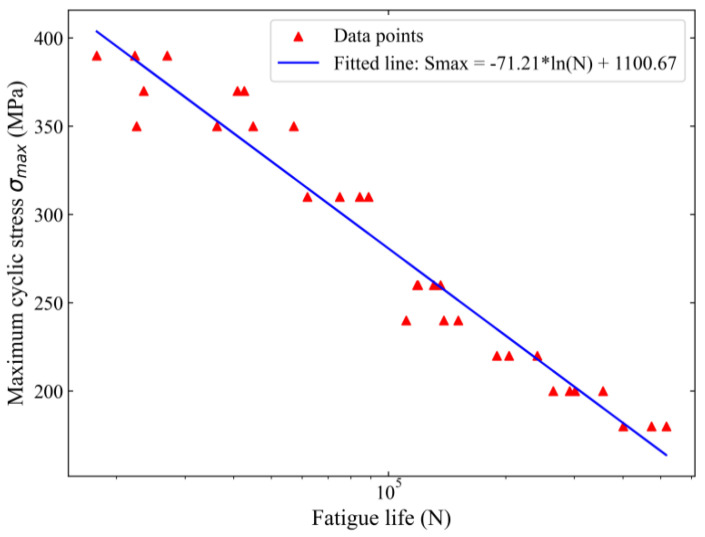
Fatigue life under the nine stress levels.

The fatigue behavior of 2024-T3 clad aluminum alloy was assessed using a Shimadzu SFL-200 kN electro-hydraulic testing system(EHF-EV200K2-040-1A, Shimadzu, Kyoto, Japan). Fatigue test data were collected via an integrated data analysis and logging platform, triggered upon detecting fatigue failure in the aluminum specimens. The evaluation parameters included sinusoidal loading, a stress ratio (*R* = 0.1), and a frequency of 20 Hz. Fatigue life results were obtained under maximum cyclic stress values *σ_max_* of 180, 200, 220, 240, 260, 310, 350, 370, and 390 MPa. For each stress level, three to four parallel experiments were conducted. The calculated results are presented in Figure 2.

In this study, the dataset was divided into three subsets—training, validation, and testing sets to evaluate the learning process and performance of the machine learning models. The training set, which accounted for approximately 70% of the total dataset, was used to train the models. The validation set, comprising around 15% of the data, was utilized during the training process to fine-tune hyperparameters and prevent overfitting. The remaining 15% of the data was allocated to the testing set, which was used to evaluate the final model performance. To ensure robustness and reduce variance in the model evaluation, five fold cross-validation was employed. This method divides the dataset into five equal folds, iteratively training the model on four folds and testing it on the remaining fold. The results are then averaged to obtain a more reliable performance metric, enhancing the generalizability of the model.

## 3. Physical Model for Fatigue Life Prediction

### 3.1. Development of an Improved SWT Equation

Revealing the fatigue failure mechanisms of 2024-T3 clad Al alloy is essential for developing accurate fatigue life prediction models. To precisely predict fatigue life, researchers have proposed various classical models. Among these, the Manson–Halford (MH) equation and the Smith Watson Topper (SWT) equation are widely used. Although the MH equation is well-suited for predicting the fatigue life of many metal materials, it does not directly account for the impact of maximum stress during cyclic loading on fatigue failure. To overcome this limitation, Smith and colleagues introduced the SWT equation [14]. They proposed that in tensile failure processes, the maximum stress in the load spectrum governs the influence of mean stress on fatigue life. For a given fatigue life, the maximum stress *σ*_max_ (strain-controlled) or the maximum strain amplitude *ε_a_* (stress-controlled) may change, but their product *σ*_max_*ε_a_* remains constant. Therefore, they used *σ*_max_*ε_a_* as the damage control parameter and developed the SWT model

(1) $$\sigma_{\max}\varepsilon_{a}={\left(\sigma_{f}^{\prime }\right)}^{2}\frac{1}{E}{\left(2N_{f}\right)}^{2b}+\sigma_{f}^{\prime }\varepsilon_{f}^{\prime }{\left(2N_{f}\right)}^{b+c}$$

where *b* is the fatigue strength exponent, *c* is the fatigue ductility exponent, *N_f_* is the fatigue life, $\sigma_{f}^{\prime }$ is the fatigue strength coefficient, and $\varepsilon_{f}^{\prime }$ is the fatigue ductility coefficient. The parameters $\sigma_{f}^{\prime }$ and $\varepsilon_{f}^{\prime }$ were obtained using the method proposed by Wang et al. [32]

(2) $$\sigma_{f}^{\prime }=\frac{\sigma_{b}}{\left(1-\delta \right){\left(0.5\right)}^{b}}$$

(3) $$\varepsilon_{f}^{\prime }=\frac{-\ln(1-\delta )}{{\left(0.5\right)}^{c}}$$

Compared to other models, the SWT model better reflects the influence of mean stress on material life. Good predictive performance is achieved for most materials. However, only the effect of maximum stress on mean stress is considered in the SWT model. The sensitivity of different materials to mean stress is not reflected. Additionally, the effect of mean stress on material plastic deformation is ignored. This results in conservative predictions for some materials. To address this issue, it was proposed by Walker that different materials exhibit varying sensitivities to mean stress [33]. A material constant *γ* was introduced and associated with the stress ratio *R* to reflect the sensitivity of different materials to mean stress. Consequently, the Walker mean stress correction criterion was proposed, which is expressed as

(4) $$\sigma_{a,eff}=\sigma_{a}{\left(1-R\right)}^{\gamma }$$

where *σ_a_* is the nominal stress amplitude and *σ_a,eff_* is the effective stress amplitude. Since the γ values for different materials must be obtained through extensive experimental data, the applicability is limited. To address this, Li et al. [34] compared the fatigue performance parameters of various materials. They found that the magnitude of *γ* is intrinsically related to the material’s yield strength and tensile strength. Additionally, when the yield strength and tensile strength are similar, *γ* approaches 0.5. Based on these findings, they proposed a formula for calculating the Walker index:

(5) $$\gamma =0.5\pm \frac{\sigma_{b}-\sigma_{s}}{\sigma_{b}+\sigma_{s}}$$

By substituting the tensile strength *σ_b_* and the yield strength *σ_s_*, the value of *γ* is determined to be 0.67.

**Figure 3:**
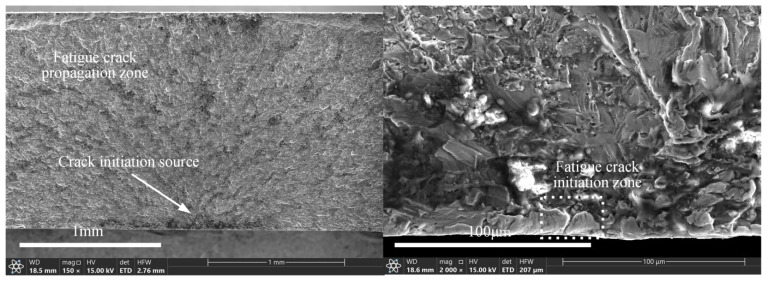
Fatigue fracture of 2024-T3 clad Al alloy.

The fatigue performance of materials is influenced by various factors, including stress concentration and surface roughness [35]. Therefore, a stress correction factor needs to be introduced to adjust the nominal stress. Figure 3 shows the fatigue fracture surface of 2024-T3 clad Al alloy under maximum cyclic stress. Scanning electron microscope (SEM) images reveal that fatigue cracks initiated on the sample surface and propagated inward at approximately a 45-degree angle. This indicates that surface roughness plays a critical role in the initiation and propagation of fatigue cracks.

**Figure 4:**
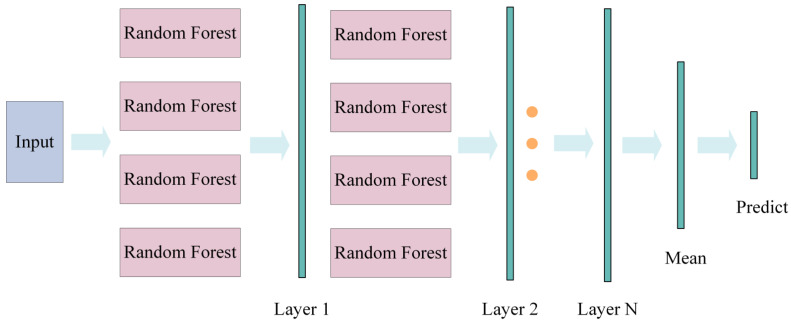
The structure of the deep Random Forest.

In addition to surface roughness, surface protrusions and depressions caused by dislocation slip are important factors contributing to stress concentration on the surface of 2024-T3 clad Al alloy. The dislocation equilibrium condition can be represented as [36]

(6) $$\tau_{1}^{D}+\tau_{1}-k=0$$

where *τ*_1_^D^ represents the back stress caused by dislocations accumulating at the boundary, and *k* denotes the frictional stress. Because the specimen has a free surface, fewer obstacles impede dislocation slip. Consequently, dislocations can more easily slip to the surface. The frictional stress *k*, grain size 2*a*′, Burgers vector *b*, and constant *A*, which is associated with the dislocation stress field, are determined by material properties. Therefore, the dislocation number *N_D_* in 2024-T3 Al alloy is influenced by the shear stress τ_1_. A larger shear stress τ_1_ allows dislocations to overcome obstacles such as grain boundaries. Consequently, more dislocations gradually slip to the specimen surface. The movement of dislocations results in the formation of intrusion and extrusion regions on the surface of the specimen. The sizes of these regions are determined by the plastic displacement *γ*_1_ ($\gamma_{1}=\pi N_{D}ba\prime /2$). The plastic displacement *γ*_1_ increases with the number of dislocations *N_D_*. As a result, the size of the intrusion zone *d*_In_ increases along the direction of maximum shear stress τ_1_. For specimens exposed to specific cyclic stresses, the surface valley depth *d’* shows a positive correlation with the intrusion size *d*_In_ (*d*_In_ = *d*′ *− d*), where *d* represents the initial valley depth. This indicates that stress concentration, arising from extrusion zones and surface valleys, promotes crack initiation on the surface of 2024-T3 clad aluminum alloy specimens. The fatigue failure mechanisms described in Figure 4 align with the failure features observed on the fracture surface. This confirms that surface roughness and dislocation slip play critical roles in stress concentration and the initiation of fatigue cracks.

These microstructural features further affect the material’s fatigue life by influencing the stress concentration factor *K_t_* and the surface roughness coefficient *C_s_*. Based on the above analysis, the nominal stress needs to be corrected. First, the theoretical stress concentration factor *K_t_* describes the local stress amplification effect caused by geometric discontinuities. Next, a notch sensitivity factor *q* is introduced to reflect the material’s sensitivity to notch geometry. The fatigue stress concentration factor *K_f_* combines *K_t_* and *q*, and is calculated as follows:

(7) $$K_{f}=1+q(K_{t}-1)$$

The theoretical stress concentration factor *K_t_* was determined to be 1.15 through finite element simulation. Additionally, the surface roughness coefficient *C_s_* accounts for the impact of surface microstructure peaks and valleys on the initiation and propagation of fatigue cracks. By integrating the aforementioned correction factors, the comprehensive correction coefficient *K_l_* is obtained:

(8) $$K_{l}=K_{f}C_{s}$$

By combining the stress concentration correction factor with the Walker equation, the comprehensively corrected stress amplitude is obtained:

(9) $$\sigma_{a}^{\prime }=K_{l}\sigma_{a}{\left(1-R\right)}^{\gamma }$$

Similarly, the corrected maximum stress is

(10) $$\sigma_{\max}^{\prime }=K_{l}\sigma_{\max}$$

According to the Ramberg Osgood equation [37], the corrected strain amplitude is expressed as the sum of elastic strain and plastic strain:

(11) $$\varepsilon_{a}=\frac{\sigma_{a}^{\prime }}{E}+{\left(\frac{\sigma_{a}^{\prime }}{K^{\prime }}\right)}^{1/n^{\prime }}$$

where *K*′ is the cyclic strength coefficient, and *n*′ is the cyclic strain hardening exponent. According to the strain fatigue analysis manual, *K*′ is 685.73 MPa and *n*′ is 0.08 [38].

The corrected stress amplitude *σ*′*_max_* and strain amplitude *ε_a_* are substituted into the SWT equation. Further simplification is performed by expressing all parameters in terms of fundamental material and geometric properties. By substituting Equations (8), (10) and (11) into Equation (1), the improved SWT equation for Al alloy is obtained:

(12) $$\begin{array}{l} \left[\left(1+q(K_{t}-1)\right)C_{s}\sigma_{\max}\right]\left[\frac{\left(1+q(K_{t}-1)\right)C_{s}\sigma_{a}{\left(1-R\right)}^{\gamma }}{E}+{\left(\frac{(1+q(K_{t}-1))C_{s}\sigma_{a}{\left(1-R\right)}^{\gamma }}{K^{\prime }}\right)}^{1/n}\right]\\ ={\left(\sigma_{f}^{\prime }\right)}^{2}\frac{1}{E}{\left(2N_{f}\right)}^{2b}+\sigma_{f}^{\prime }\varepsilon_{f}^{\prime }{\left(2N_{f}\right)}^{b+c} \end{array}$$

### 3.2. The Improved SWT Equation Parameter Optimization

In the previous section, the improved SWT equation, as shown in Equation (12), was thoroughly derived. The key material parameters *q*, *b*, and *c* require specific values. However, traditional experimental methods for obtaining these parameters involve a significant workload and low accuracy, making them unsuitable for practical applications. To overcome these challenges, machine learning algorithms were employed to achieve efficient and precise parameter determination. Specifically, the predictive performance of various machine learning models was evaluated. The best-performing models were selected to construct a blending model. These selected models included extreme gradient boosting (XGBoost) and its derivatives, as well as Random Forest (RF) and its derivatives. Finally, the L-BFGS algorithm was integrated with the established machine learning model to solve for the multi-parameter in the improved SWT equation.

The predictive performance of different machine learning models was assessed using the Mean Absolute Percentage Error (*MAPE*) and the determination coefficient (*R*^2^). An *R*^2^ value nearing unity signifies a strong correspondence between predicted and actual measurements, indicating enhanced model precision. *MAPE* normalizes each prediction’s error, providing reliable indicators of model performance. Lower *MAPE* values reflect higher accuracy in predictions. The equations for computing *R*^2^ and *MAPE* are provided in Equations (13) and (14)

(13) $$R^{2}=1-\frac{\overset{i=1}{\underset{n}{\sum }}{\left(y_{i}-{\hat{y}}_{i}\right)}^{2}}{\overset{i=1}{\underset{n}{\sum }}{\left(y_{i}-\bar{y}\right)}^{2}}$$

(14) $$MAPE=\frac{1}{n}\overset{i=1}{\underset{n}{\sum }}\left|\frac{y_{i}-{\hat{y}}_{i}}{y_{i}}\right|\times 100\%$$

where *y_i_* denotes the observed values, ${\hat{y}}_{i}$ represents the predicted values, $\bar{y}$ is the average of the observed values, and *n* indicates the total number of samples.

XGBoost and its derivative models, as well as RF and its derivative models, were selected for predicting the fatigue life of aluminum alloy standard components. This selection was primarily based on their unique advantages in handling complex engineering datasets and performing regression and classification tasks [39]. These models exhibit complementary characteristics in addressing specific challenges of fatigue life prediction, such as nonlinear relationships, data variability, and the need for robust generalization. XGBoost is a powerful gradient-boosting framework widely applied due to its ability to model nonlinear interactions between variables, scalability, and superior performance on tabular data [40]. Predicting fatigue life requires capturing intricate nonlinear interactions among stress parameters, material properties, and environmental factors. These relationships are effectively captured by XGBoost through the integration of gradient-boosted decision trees. RF is a robust ensemble learning method that performs excellently in handling noisy or incomplete data [41]. This feature is particularly important in fatigue experiments, where measurement errors or missing values are common in experimental data. By averaging the predictions of multiple decision trees, RF reduces model variance and enhances generalization performance. This characteristic is especially important in fatigue life prediction, where variability in material properties and testing conditions often leads to data uncertainty. The combination of these two types of models ensures high prediction accuracy and strong generalization capability. The ability of XGBoost to capture complex interactions complements the advantages of RF in mitigating overfitting and handling noisy data. Together, they form a comprehensive modeling approach capable of effectively addressing the complexity of aluminum alloy fatigue life prediction and improving prediction accuracy.

Since both XGBoost and RF contain a large number of parameters, which are often discrete and non-differentiable, a genetic algorithm (GA) was employed to select and optimize these parameters. In the algorithm design, the mutation rate was set to $\frac{1}{Lind}$, where *Lind* is the chromosome length. Binary encoding was used for the representation of chromosomes. The crossover rate was established at 0.9, the population size was set to 50, and the number of iterations was fixed at 100. For the selection strategy, ranking fitness and tournament selection methods were adopted to ensure that the algorithm could effectively search for the optimal solution.

The objective function of XGBoost is defined as follows:

(15) Obj(θ)=L(θ)+Ω(θ)

where *L*(*θ*) is the loss function and Ω(*θ*) is the regularization term. Through a second-order Taylor expansion, the objective function is further transformed into

(16) $$L^{\left(m\right)}=\overset{i=1}{\underset{N}{\sum }}\left[L(y_{i},{\hat{y}}_{i}^{\left(m-1\right)})+g_{i}f_{m}(x_{i})+\frac{1}{2}h_{i}f_{m}^{2}(x_{i})\right]+\gamma J+\frac{1}{2}\lambda \overset{j=1}{\underset{J}{\sum }}b_{j}^{2}$$

where $g_{i}=\frac{\partial L(y_{i},{\hat{y}}_{i}^{m-1})}{\partial {\hat{y}}^{\left(m-1\right)}},\;h_{i}=\frac{\partial^{2}L(y_{i},{\hat{y}}_{i}^{\left(m-1\right)})}{{\left(\partial {\hat{y}}^{\left(m-1\right)}\right)}^{2}}$, *J* represents the number of leaf nodes, and *b_j_* denotes the prediction value of the leaf node. The second-order Taylor expansion requires the loss function to be twice differentiable. While XGBoost’s default loss function is the mean squared error (MSE), which satisfies this requirement, our target loss function is *MAPE*, which is not differentiable. This non-differentiability prevents the model from functioning correctly. To address this issue, we adopt the Huber loss function, defined as

(17) $$L_{\delta }(y,f(x))=\left\{\begin{array}{lll} \frac{1}{2}{\left(y-f(x)\right)}^{2} & & \mathrm{for}|y-f(x)|\leq \delta \\ \delta |y-f(x)|-\frac{1}{2}\delta^{2} & & \mathrm{otherwise} \end{array}\right.$$

Nevertheless, the Huber loss is still not entirely differentiable. Therefore, a new pseudo-Huber loss function is proposed, defined as

(18) $$L_{\delta }\left(y_{i},{\tilde{y}}_{i}\right)=\delta^{2}\left(\sqrt{1+{\left(\frac{{\tilde{y}}_{i}-y_{i}}{\delta }\right)}^{2}}-1\right)$$

where ${\tilde{y}}_{i}$ is a prediction, and $y_{i}$ is the truth. Typically, the first- and second-order derivatives of the function are calculated as follows:

(19) $$\frac{\partial }{\partial x}\left(\delta^{2}\sqrt{1+\frac{x^{2}}{\delta^{2}}}-1\right)=\frac{x}{\sqrt{\frac{x^{2}}{\delta^{2}}+1}}$$

(20) $$\frac{\partial^{2}}{\partial x^{2}}\left(\delta^{2}\sqrt{1+\frac{x^{2}}{\delta^{2}}}-1\right)=\frac{1}{{\left(1+\frac{x^{2}}{\delta^{2}}\right)}^{\frac{3}{2}}}$$

where $x={\tilde{y}}_{i}-y_{i}$. Consequently, we define the XGBoost model using the pseudo-Huber loss function as HL-XGBoost. Subsequently, the model was optimized for hyperparameters using a genetic algorithm (GA), resulting in the GA-HL-XGBoost model. The specific parameter settings are as follows: a learning rate of 0.1, a maximum depth of 6, a subsample ratio of 0.8, a column sampling ratio of 0.8, and a regularization parameter of 1. The dataset was individually trained using XGBoost, GA-XGBoost, HL-XGBoost, and GA-HL-XGBoost, and the models were subsequently evaluated. As shown in Table 3, GA-HL-XGBoost outperformed the other individual models, achieving a *MAPE* of 9.76% and an *R*^2^ of 0.93.

Subsequently, the Random Forest model was employed. Random Forest, an ensemble learning method, constructs multiple decision trees using bootstrap sampling and out-of-bag estimates to enhance the model’s robustness and generalization capabilities. However, in this study, the performance of a single Random Forest model was not optimal. To improve its performance, a genetic algorithm (GA) was employed to optimize the hyperparameters of Random Forest, resulting in the GA-RF model. In the GA-RF model, the number of trees was set to 100, the maximum tree depth was set to 10, the minimum sample count for splitting an internal node was configured to 2, and at least one sample was required for a leaf node.

Additionally, a deep Random Forest (DRF) model was developed. The deep Random Forest (DRF) model uses a cascading structure, as shown in Figure 4. Input data are processed layer by layer to optimize feature representation. First, the input data are fed into the first layer of Random Forest. After training, preliminary predictions and feature representations are generated. These features include the original input data, probability distributions, and feature importances learned by the first layer. Next, the combined features are passed to the second layer of Random Forest. This layer performs higher-order feature interactions and pattern learning. The output features from the second layer are then sent to the next layer. This process continues until the final layer of Random Forest produces the ultimate prediction results. The model consists of four fixed layers. Each layer contains 200 trees. The maximum depth of each tree is set to 12. This setting ensures that complex patterns are captured while controlling computational complexity and maintaining generalization ability. A layer-by-layer feature fusion strategy is used to process input features. The input features for each layer include the probability distributions from the previous layer and the original input features. This design maintains the integrity of the original information and gradually enhances the nonlinear feature representation capability. During model training, a layer-wise accuracy monitoring criterion is implemented. After training each layer, the prediction accuracy is evaluated using 10-fold cross-validation. If the accuracy improvement of a layer is less than 0.5%, training is terminated early. This prevents unnecessary computational overhead and overfitting. The model shows excellent performance in complex feature extraction and significantly improves prediction accuracy.

To fully leverage the advantages of each model, a blending method was employed to combine the prediction results of three models: GA-HL-XGBoost, GA-RF, and DRF. The combined prediction is defined as

(21) $$f(x)=\overset{n}{\sum }\alpha_{i}f_{i}(x)$$

(22) $$s.t\sum \alpha_{i}=1$$

where $\alpha_{i}$ is the weight of the different models, and $f_{i}(x)$ is the model prediction results. The weights were determined and optimized using a genetic algorithm to ensure the optimal overall predictive performance of the ensemble model. The weights for the optimal overall predictive performance of the blending model are

(23) f(x)=0.31GA-HL-XGBoost+0.22GA-RF+0.47DRF

**Figure 5:**
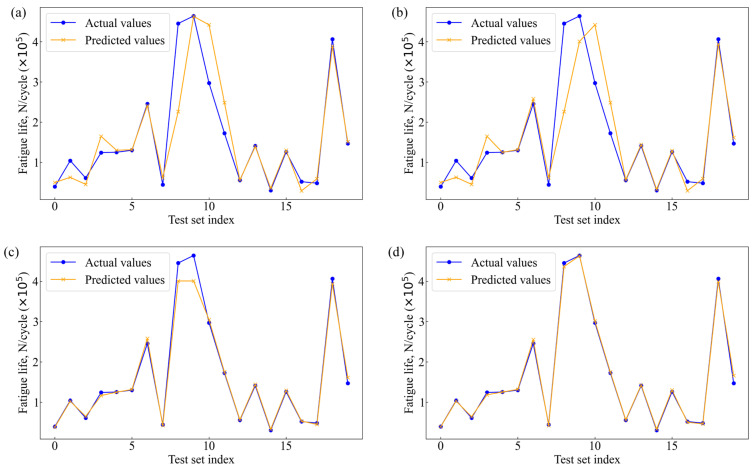
Comparison of actual and predicted values: (**a**) GA-HL-XGBoost, (**b**) GA-RF, (**c**) DFR, and (**d**) blending.

The fatigue life of 2024-T3 clad Al alloy was estimated using the blending model along with its constituent models. Figure 5 demonstrates the effectiveness of these machine learning models in forecasting fatigue life. The specific parameter settings for these algorithms are provided in Appendix A. Figure 5a displays the prediction results of the GA-HL-XGBoost model. This model demonstrates a relatively high prediction accuracy, capturing the general trend of the fatigue life data. However, there are slight deviations in regions with extreme fatigue life values, indicating areas where the model’s predictive capability could be further enhanced. The GA-HL-XGBoost model benefits from the robust gradient boosting framework enhanced by genetic algorithm optimization, which improves feature selection and hyperparameter tuning, thereby enhancing its ability to model complex nonlinear relationships. In Figure 5b, the performance of the GA-RF model is presented. The GA-RF model shows a good alignment with the actual fatigue life values, particularly in the mid-range cycles. Nonetheless, similar to the GA-HL-XGBoost model, it exhibits noticeable discrepancies at the higher and lower extremes of the fatigue life spectrum. This suggests that while the GA-RF model effectively captures the overall data patterns through ensemble learning, it may struggle with extreme values due to limitations in handling highly nonlinear variations inherent in the data. Figure 5c illustrates the prediction accuracy of the DRF model. The DRF model provides a balanced prediction across the entire range of fatigue life values, showing fewer deviations compared to the GA-RF model. The deep representation capabilities of the DRF model contribute to its improved performance in capturing intricate data patterns. However, minor prediction errors are still observable, particularly in regions with rapidly changing fatigue life values, indicating room for further refinement. The blending model’s performance is depicted in Figure 5d. This model integrates the strengths of GA-HL-XGBoost, GA-RF, and DRF, resulting in superior prediction accuracy compared to its individual constituents. The blending model exhibits a high degree of overlap between the predicted values and the actual fatigue life measurements across all sample indices, demonstrating its enhanced ability to accurately predict fatigue life. The blending approach effectively mitigates the individual weaknesses of each model, providing a more robust and generalized prediction capability. This is particularly evident in the reduction in prediction errors in regions where individual models previously showed deviations.

**Figure 6:**
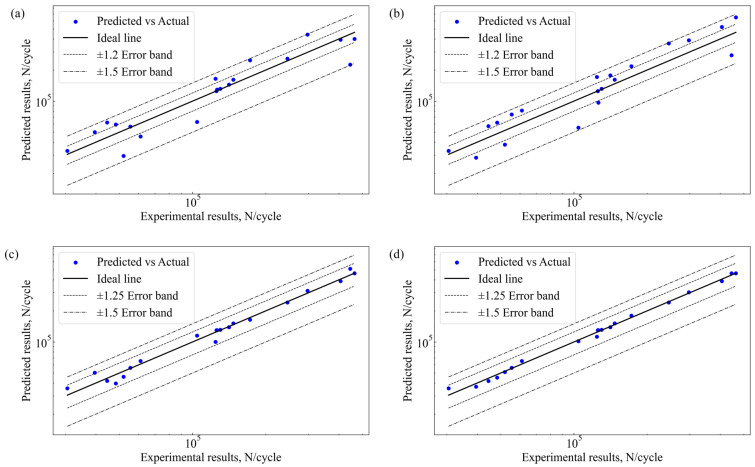
Fatigue life predictions vs. experimental results: (**a**) GA-HL-XGBoost, (**b**) GA-RF, (**c**) DFR, and (**d**) blending.

Figure 6 illustrates the quantitative relationship between the predicted and actual fatigue life values for 2024-T3 clad Al alloy. Figure 6a shows the prediction results of the GA-HL-XGBoost model. Most predicted values fall within the ±1.25 error band, indicating high prediction accuracy in most cases. However, some data points exceed the ±1.25 error band, especially in regions with high fatigue life values. This suggests that the model has some prediction errors when handling extreme values. This may be due to the model not fully capturing certain complex nonlinear relationships during feature selection and hyperparameter tuning. Figure 6b presents the prediction results of the GA-RF model. Most predicted values are concentrated within the ±1.5 error band, indicating relatively large prediction errors, particularly in regions with extreme fatigue life values. Although the GA-RF model improves overall prediction ability through ensemble learning with multiple decision trees, its accuracy is still limited when dealing with highly nonlinear and complex data distributions. This highlights the challenges of parameter optimization and the influence of nonlinear feature distributions within high-dimensional spaces on model accuracy. Figure 6c illustrates the prediction accuracy of the DRF model. Similar to the GA-RF model, most predicted values fall within the ±1.5 error band. However, compared to the GA-RF model, the DRF model has more data points within the ±1.25 error band, showing some improvement. The deep representation capability of the DRF model helps capture more complex data patterns. Nevertheless, a few data points still lie outside the error bands in regions with rapidly changing fatigue life values, indicating that the model’s prediction ability in these areas can be further enhanced. Figure 6d shows the prediction results of the blended model. The blending model integrates the predictions of GA-HL-XGBoost, GA-RF, and DRF, significantly improving overall prediction performance. Most predicted values lie within the ±1.25 error band, and very few data points exceed the ±1.25 error band. This demonstrates that the blended model has higher accuracy and robustness in handling complex nonlinear data. The blending approach effectively combines the strengths of each individual model, compensating for their respective weaknesses and maintaining high prediction accuracy across different fatigue life ranges.

To comprehensively evaluate the models’ performance, the *R*^2^ and *MAPE* metrics were computed. The performance results are summarized in Table 4, offering a thorough quantitative analysis of each model’s predictive capability for the fatigue life of 2024-T3 clad aluminum alloy. The GA-HL-XGBoost model achieved an *R*^2^ value of 0.93 and a *MAPE* of 9.76%, demonstrating a strong agreement between the predicted and actual fatigue life values, along with a relatively low average prediction error. The GA-RF model obtained an *R*^2^ of 0.92 and a *MAPE* of 10.11%, which are slightly lower than those of the GA-HL-XGBoost model. This suggests that while the GA-RF model has high explanatory power, its prediction accuracy is marginally reduced compared to GA-HL-XGBoost. The DRF model demonstrated the highest *R*^2^ of 0.94 among the individual models, coupled with a *MAPE* of 8.98%. These results indicate that the DRF model has excellent fitting capability and superior prediction accuracy, effectively capturing the complex patterns and nonlinear relationships in the data. The blending model outperformed all individual models, achieving an *R*^2^ of 0.96 and a *MAPE* of 7.45%. The highest *R*^2^ signifies that the blended model explains 96% of the variance in the fatigue life data, while the lowest *MAPE* reflects its superior accuracy in minimizing the average prediction error. This enhanced performance is attributed to the blending method, which integrates the strengths of GA-HL-XGBoost, GA-RF, and DRF, thereby compensating for their individual limitations and achieving a more robust and reliable prediction capability.

In summary, while the GA-HL-XGBoost, GA-RF, and DRF models each show certain predictive capabilities, the blending model significantly enhances the prediction accuracy and stability for the fatigue life of 2024-T3 clad Al alloy by leveraging the strengths of multiple models.

The blending model was selected as the most suitable approach for predicting the fatigue life. Subsequently, the parameters *q*, *b*, and *c* within the physical model were determined based on the predicted values obtained from the model. Initially, the blended model was utilized to estimate the fatigue life, and the corresponding prediction results were recorded. Then, the L-BFGS-B algorithm was applied to optimize the parameters of the physical model using the blended model’s outputs. The L-BFGS-B algorithm is a computational method designed for solving large-scale optimization problems [42]. By applying the L-BFGS-B algorithm to Equation (12), the parameter values *q*, *b*, and *c* were found to be 0.82, −0.095, and −0.69, respectively. Reference to the strain fatigue analysis handbook indicates that the obtained parameter values are all within reasonable ranges [38].

Therefore, the parameter values derived in this study are both precise and reliable. Moreover, the proposed parameter-solving approach used to predict fatigue life is validated. Finally, a robust physical equation for estimating the fatigue life was established.

## 4. Numerical Solution of the Fatigue Life Prediction Equation

In previous sections, an improved SWT fatigue life prediction model was developed to predict the fatigue behavior of 2024-T3 clad Al alloy. However, the highly nonlinear nature of the SWT equation complicates its solution. Traditional numerical methods, such as the Newton Raphson iterative method, are highly sensitive to initial values, have slow convergence rates, and are prone to becoming trapped in local optima. Additionally, in cases of complex material properties or varying stress conditions, traditional methods fail to meet the efficiency and robustness requirements of engineering applications. Therefore, reinforcement learning methods were introduced to solve nonlinear equations. With their adaptive learning capabilities and dynamic optimization strategies, they can effectively address complex nonlinear problems. This study utilizes the deep deterministic policy gradient (DDPG) algorithm combined with the Newton iteration method to solve the improved SWT equation in a reinforcement learning environment. An efficient, stable, and accurate fatigue life prediction scheme was thus proposed.

### 4.1. DDPG Algorithm and Model Construction

The deep deterministic policy gradient (DDPG) is a reinforcement learning algorithm. It is designed for optimization problems with continuous action spaces [43]. The DDPG combines the strengths of deep Q-Networks (DQN) and policy gradient methods. This combination allows the DDPG to make dynamic decisions in complex nonlinear environments. It can directly generate continuous actions. The value network evaluates the quality of these actions. This process optimizes the strategy and improves solving efficiency. In the DDPG algorithm, an agent learns by interacting with the environment. The algorithm is based on the Markov Decision Process (MDP). The agent aims to maximize the cumulative reward *R*. The policy network $\mu (s|\theta^{\mu })$. takes the current state st as input. It outputs the action at to control the dynamic system. The value network $Q(s,a|\theta^{Q})$ assesses the quality of the current state–action pair. This assessment guides the optimization of the policy network. To enhance learning stability, the DDPG uses target networks and an experience replay mechanism. These features enable efficient training of both the policy and value networks.

A key feature of the DDPG is its ability to handle continuous action spaces. Traditional reinforcement learning algorithms, such as Q-learning or DQN, use discrete action spaces. Discretization can cause accuracy loss. The DDPG optimizes directly in continuous state–action spaces. It generates continuous actions through the policy network. This avoids the limitations of discretization. This capability is suitable for solving the improved SWT equation. The SWT equation has complex nonlinear characteristics. Solving it requires dynamic adjustment of step size factors. In solving the improved SWT equation, the state space is defined by the current estimated fatigue life *N_f_* and the equation error *F*(*N_f_*). This design reflects the current solving process. It provides sufficient information for the agent’s strategy optimization. The action space is defined as the step size adjustment value Δ*λ*. The range is set to [−1.0, 1.0]. This dynamic adjustment allows the agent to flexibly modify variables. It balances convergence speed and solution accuracy. The reward function is crucial in the DDPG. Its design directly affects the agent’s learning efficiency and strategy optimization. In this study, the reward function considers error, fatigue life deviation, and iteration cost

(24) $$R=-\log_{10}(|F(N_{f})|+\epsilon )-\beta \log_{10}(|N_{f}-N_{f,true}|+\epsilon )-\alpha \times n$$

In the reward function, *F*(*N_f_*) represents the current error of the improved SWT equation. It is the core metric that needs to be optimized. ϵ is a small value to prevent numerical issues. *N_f_*_,true_ is the true fatigue life value. *β* and *α* are weight factors. They balance error optimization and computational cost. n is the current iteration count. It reflects the computational cost. This reward mechanism guides the agent to quickly reduce errors and approach the true solution. At each time step, the agent generates an action Δ*λ* through the policy network. It updates the fatigue life estimate using the Newton iteration method:

(25) $$N_{f}^{t+1}=N_{f}^{t}-\frac{F(N_{f}^{t})}{F^{\prime }(N_{f}^{t})}+\Delta \lambda$$

where $F(N_{f}^{t})$ is the current error of the estimated fatigue life. $F^{\prime }(N_{f}^{t})$ is the derivative of the error with respect to the fatigue life estimate. It indicates the correction direction. This design integrates the exploration ability of reinforcement learning with the efficient convergence of traditional numerical methods. The stability of the DDPG is greatly enhanced by the introduction of target networks. The target network is a slowly changing copy of the main network. It uses a soft update strategy to gradually approach the main network. The parameter update formula is

(26) $$\theta_{\mathrm{target}}\leftarrow \tau \theta +(1-\tau )\theta_{\mathrm{target}}$$

where *τ* is the update rate parameter. It is typically in the range of [0.001, 0.01]. The use of target networks prevents instability caused by drastic updates in the main network. Additionally, the experience replay mechanism stores the agent’s interactions with the environment as state–action–reward–next state tuples. Training is performed through random sampling. This significantly reduces temporal correlations between samples. It improves data utilization efficiency and model generalization. To further enhance solution accuracy, the DDPG incorporates a noise mechanism. It adds random perturbations, such as Ornstein–Uhlenbeck noise, to actions. This increases the agent’s exploration capability in the early training stages. In later stages, the noise gradually decreases. The agent shifts from exploration to exploitation. This improves strategy stability and precision.

### 4.2. Model Training and Result Analysis

To validate the effectiveness of the DDPG in solving the improved SWT equation, the model was systematically trained and tested in this study. The specific parameter settings for the Deep Deterministic Policy Gradient (DDPG) algorithm are provided in Appendix B. The training results were comprehensively analyzed using various evaluation charts. The training process focused on the agent learning the optimal strategy through interactions with the environment, thereby gradually enhancing the efficiency and accuracy of the solution.

**Figure 7:**
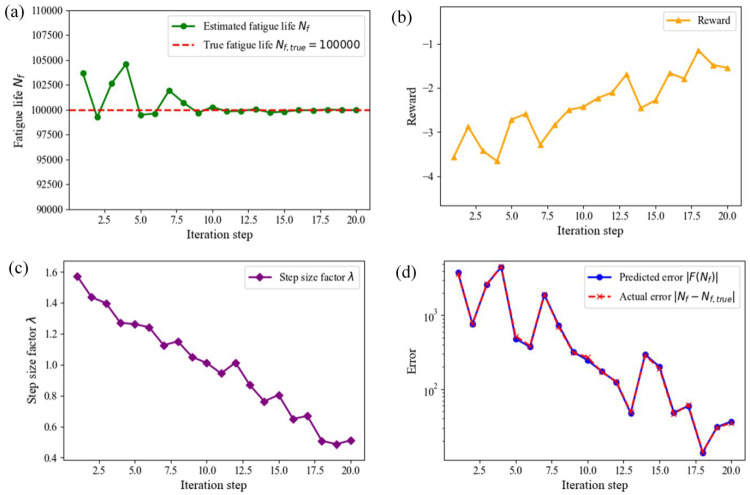
Model training results. (**a**) fatigue life convergence trajectory, (**b**) reward value change curve, (**c**) step size factor dynamic adjustment trajectory, and (**d**) comparison of prediction error and actual error.

Figure 7 presents the training results of the model, including the fatigue life convergence trajectory, error convergence trend, changes in reward values, and the dynamic process of step size factor adjustments. Figure 7a shows the convergence trajectory of fatigue life *N_f_*. This graph assesses the dynamic changes in the agent’s predicted fatigue life. The *x*-axis represents the iteration steps, and the *y*-axis represents the fatigue life estimate *N_f_*. The green curve shows the agent’s predicted values at each iteration step. The red dashed line represents the true value *N_f_*_,true_. In the initial stage, the agent’s predictions deviated significantly from the true value and exhibited large fluctuations. This indicates that the agent was in the exploration phase, learning from environmental feedback to optimize its strategy. As training progressed, the predictions stabilized and approached the true value. This reflects the model’s convergence and efficiency. The gradual reduction in fluctuation amplitude indicates that the agent adopted more precise step size adjustment strategies in the later stages, thereby improving prediction accuracy. Figure 7b depicts the trend of reward value changes during the agent’s training process. The *x*-axis represents iteration steps, and the *y*-axis represents reward values. The reward values generally show an upward trend, increasing from a low level in the initial stage. This indicates that the agent’s learning effectiveness improves as training progresses. In the early stages, reward values fluctuated significantly due to large errors and the model not yet fully learning. As the agent gradually reduced errors and optimized predictions, reward values stabilized. The fluctuations in reward values reflect the agent’s dynamic adjustment capability while balancing exploration and exploitation, verifying the effectiveness of the designed reward function. Figure 7c shows the variation in the step size factor *λ* with iteration steps. The *x*-axis represents iteration steps, and the *y*-axis represents the value of the step size factor. In the initial stage, the step size factor was large, which helped the agent quickly explore the possible solution space. As iterations progressed, the step size factor gradually decreased from an initial value of 1.6 to approximately 0.6. The decreasing trend of the step size factor indicates that the agent made finer adjustments as it approached the true solution, avoiding oscillations caused by excessive corrections. The dynamic step size adjustment strategy significantly improved the model’s convergence speed and solution accuracy. Figure 7d presents the comparison curves of prediction error |*F*(*N_f_*)| and actual error |*N_f_* − *N_f_*_,true_|. The *x*-axis represents iteration steps, and the *y*-axis represents error values, using a logarithmic scale to clearly show the error reduction process. The blue curve represents the prediction error, and the red curve represents the actual error. The two curves closely match, indicating that the model can accurately assess its own errors and demonstrating the agent’s adaptive learning capability. In the early iterations, the errors decreased rapidly, showing the model’s efficient learning ability in the initial stage. In later iterations, the errors stabilized, further verifying the model’s solution accuracy and convergence.

Comprehensive analysis of the four sub-figures shows that the DDPG algorithm exhibits good convergence, efficiency, and robustness in solving the improved SWT equation’s nonlinear problem. The agent achieved rapid convergence and high-precision solutions through dynamic adjustment of the step size factor. The gradual increase in reward values verified the model’s learning capability. The exponential decrease in errors and the dynamic optimization of the step size factor further supported the scientific validity and practicality of this method. This indicates that the reinforcement learning method based on the DDPG can efficiently handle complex nonlinear problems. It provides an innovative solution for fatigue life prediction.

### 4.3. Model Accuracy Validation

**Figure 8:**
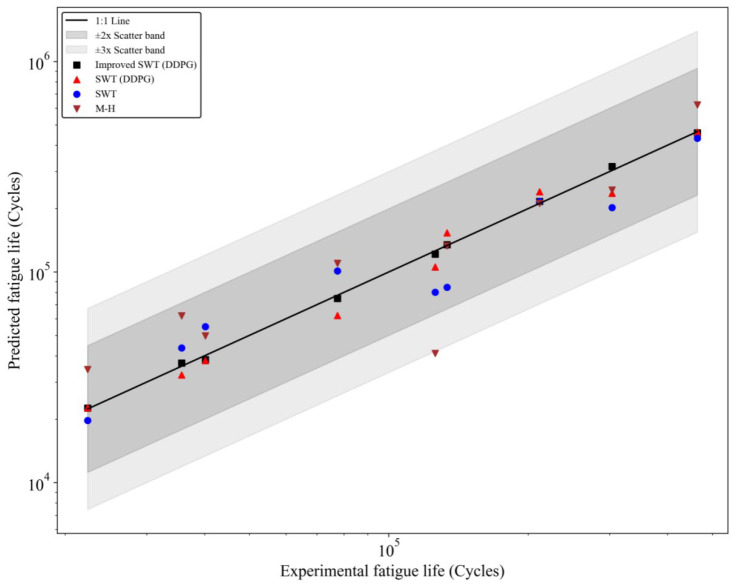
Comparison of experimental and predicted fatigue life.

To evaluate the predictive capability of the improved SWT (combined with the DDPG) model and its advantages in fatigue life prediction, it was compared with three other models: the traditional SWT model (solved using the Newton Raphson method for nonlinear equations), the traditional SWT model combined with the DDPG (without improvements), and the Manson Halford (M-H) model. The main basis for model performance comparison was the agreement between experimental data and model predictions and their distribution patterns. The analysis results are shown in Figure 8. Figure 8 compares experimental fatigue life with predicted fatigue life. The *x*-axis represents experimental fatigue life (in cycles), and the *y*-axis represents model-predicted fatigue life (in cycles). A logarithmic scale is used for a clear presentation of prediction accuracy. The black solid line is the ideal 1:1 reference line, indicating perfect agreement between predicted and experimental fatigue life. The gray regions represent the ±2 and ±3 spread bands, reflecting the allowable deviation range between model predictions and experimental values. Different models are represented by different symbols in the figure: the improved SWT (DDPG) model is shown by black squares, the traditional SWT model combined with the DDPG is shown by red upward triangles, the traditional SWT model is shown by blue circles, and the M-H model is shown by brown downward triangles.

The prediction points of the improved SWT (DDPG) model all fall within the ±2 spread band and are closely distributed near the 1:1 reference line. This indicates excellent prediction accuracy and stability. This performance results from several innovations in the model. First, the improved SWT model modifies the stress concentration effect *K_t_* and the surface roughness coefficient *C_s_*, thus more comprehensively considering the microscopic mechanisms of fatigue crack initiation and propagation. Additionally, by combining the DDPG algorithm, the model uses dynamically adjusted step size factors when solving nonlinear equations. This significantly improves solution accuracy and convergence speed, especially in complex fatigue life prediction tasks in high-stress areas. The traditional SWT model combined with the DDPG, although slightly less accurate than the improved SWT model, still outperforms the traditional SWT and M-H models. Its prediction points close to the 1:1 reference line. Unlike the traditional SWT model, the traditional SWT model combined with the DDPG dynamically optimizes the step size factor *λ* during the solving process using reinforcement learning. This mitigates the high sensitivity to initial values and potential divergence issues of the Newton Raphson method. However, since this model does not adjust for the stress concentration effect and surface roughness in fatigue life, its predictions in high-stress areas show some deviations. This indicates that while the reinforcement learning solving advantage is significant, the model’s inherent limitations still restrict its application effectiveness. The prediction points of the traditional SWT model are more scattered. This is mainly because the traditional SWT model does not adjust for the stress concentration effect *K_t_* and surface roughness *C_s_*. As a result, it cannot accurately represent fatigue crack propagation behavior under complex stress conditions. Furthermore, the Newton Raphson method used for solving nonlinear equations is highly sensitive to initial values and lacks an intelligent adjustment mechanism. This makes it prone to convergence difficulties or error accumulation, which further weakens the model’s predictive performance. The M-H model has the most dispersed prediction points. This is because the M-H model is an empirical fatigue life prediction method that does not fully consider the effects of stress concentration, surface roughness, and plastic deformation on fatigue crack propagation. Additionally, compared to other models, the M-H model does not accurately characterize the material’s nonlinear behavior. This leads to significant prediction errors in both high-stress and low-stress areas.

By comparing the prediction results of the four models, it is evident that the improved SWT model (combined with the DDPG) performs the best in terms of prediction accuracy and stability. Its distribution dispersion is minimal, significantly outperforming the other three models. This result indicates that the improved SWT model enhances the ability to describe complex fatigue life-influencing factors by introducing correction factors such as stress concentration effects, surface roughness, and size effects. Additionally, the incorporation of the reinforcement learning algorithm (DDPG) provides an intelligent dynamic optimization strategy for solving nonlinear equations. This ensures that the model maintains high prediction accuracy and stability even under high-stress areas and complex loading conditions. The comparative study not only validates the superiority of the improved SWT model combined with the DDPG but also highlights the significant value of reinforcement learning algorithms in solving complex nonlinear equations. Specifically, in fatigue life prediction tasks, the DDPG’s dynamic learning capability allows the model to achieve a good balance between optimization speed and solution accuracy, providing important directions for future research.

**Figure 9:**
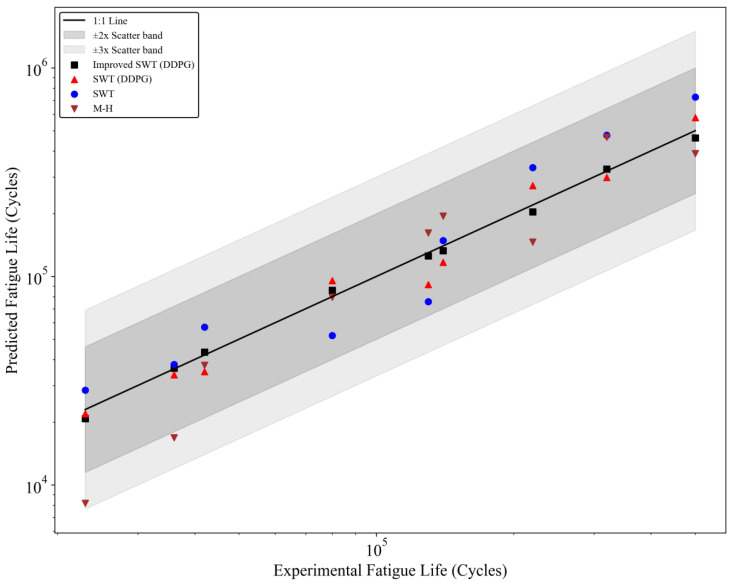
Fatigue life prediction results for GH909.

**Figure 10:**
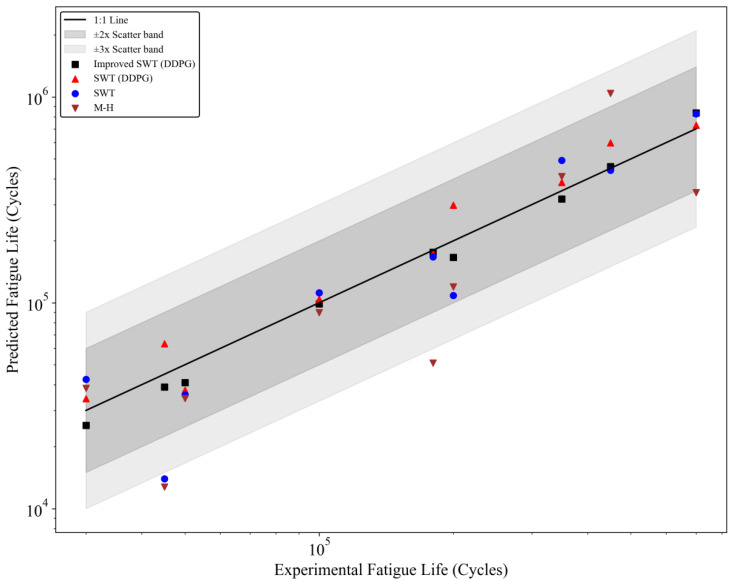
Fatigue life prediction results for TA11.

To further investigate the effectiveness of the improved SWT model (combined with the DDPG) in predicting the fatigue life of other aerospace engine materials, the stress and fatigue life data of GH909 and TA11 alloys, as measured experimentally in reference [38], were used to validate the prediction accuracy and applicability of the improved model. The material parameters for GH909 and TA11 alloys are listed in Table 5, where the alloy parameters *q*, *b*, and *c* are obtained using the parameter-solving method mentioned in the previous section. The fatigue life prediction results for GH909 and TA11 alloys are shown in Figure 9 and Figure 10, respectively.

As shown in Figure 9 and Figure 10, the fatigue life prediction results for GH909 and TA11 alloys based on the improved SWT model (combined with the DDPG) are both within the ±2 scatter band and are close to the 45-degree line, clearly outperforming other prediction models. This indicates that the new model demonstrates superior predictive capability for different aerospace materials. The method exhibits good accuracy and generalizability for predicting the fatigue life of aerospace alloy materials.

The improved SWT model (combined with the DDPG) proposed in this study not only comprehensively considers key influencing factors in theoretical analysis but also significantly enhances the model’s applicability and robustness by combining it with intelligent optimization algorithms. The successful application in predicting the fatigue life of 2024-T3 clad Al alloy further demonstrates the method’s effectiveness and engineering significance. This provides a reliable scientific basis for aerospace material design and fatigue life assessment.

## 5. Conclusions

(1) An improved SWT model was developed by incorporating mean stress effects, stress concentration factors *K_t_*, and surface roughness coefficients *C_s_*. These modifications refine stress amplitude calculations and address critical factors influencing fatigue behavior. The model integrates material constants such as fatigue strength coefficients, ductility coefficients, and elastic modulus to achieve higher prediction accuracy.
(2) An improved SWT equation to predict the fatigue life of 2024-T3 clad Al alloy is proposed. A blending model was constructed using XGBoost, RF, and their derivative models. The blending model not only predicted the fatigue life but also, in conjunction with the L-BFGS algorithm, determined the key parameters of the improved SWT physical model. The determined parameters showed strong agreement with existing experimental data. These results confirm that the proposed fatigue life prediction model is reasonable and reliable.
(3) The effectiveness of integrating reinforcement learning (DDPG algorithm) in solving the nonlinear equations of the improved SWT model is demonstrated. The dynamic optimization strategy provided by the DDPG ensured efficient convergence and effectively solved nonlinear equations in fatigue life prediction tasks. By dynamically adjusting the learning strategies during the solution process, the model achieved high prediction accuracy and stability under various loading conditions. The improved SWT prediction model combined with the DDPG showed excellent accuracy, providing a reliable solution for fatigue life prediction.
(4) Future research could focus on expanding the dataset by incorporating more diverse experimental conditions to enhance the model’s robustness. Additionally, integrating multi-physics simulations, such as temperature and stress fields, may improve prediction accuracy. Optimizing machine learning algorithms and exploring real-time monitoring techniques for dynamic fatigue life prediction could further advance the practical application of this model in industries like aerospace and automotive.

## Figures and Tables

**Table 1:** Chemical composition of 2024-T3 clad Al alloy (wt.%).

	Element	Cu	Si	Fe	Mn	Mg	Zn	Cr	Ti	Other	Al
Content											
2024-T3	Core	3.8~4.9	0.5	0.5	0.3~0.9	1.2~1.8	0.25	0.1	0.15	0.15	Other
	Cladding	0.1	≤0.7		0.05	0.05	0.1	-	0.03	-	≥99.3

**Table 2:** Mechanical properties of 2024-T3 clad Al alloy.

Elastic Modulus *E* (GPa)	Tensile Strength *σ_b_* (MPa)	Yield Strength *σ_S_* (MPa)	Elongation *δ* (%)
72.2	456	326	23.8

**Table 3:** Comparison of prediction accuracy for different models.

ML Model	*R^2^*	*MAPE* (%)
XGBoost	0.88	13.43
GA-XGBoost	0.90	11.45
HL-XGBoost	0.91	12.87
GA-HL-XGBoost	0.93	9.76

**Table 4:** Comparison of prediction accuracy for different models.

ML Model	*R^2^*	*MAPE* (%)
GA-HL-XGBoost	0.93	9.76
GA-RF	0.92	10.11
DRF	0.94	8.98
Blending	0.96	7.45

**Table 5:** Material parameters of GH909 and TA11 alloys.

Material	E (GPa)	σ_b_ (MPa)	σ_S_ (MPa)	q	b	c
GH909	160	1104	872	0.31	−0.064	−0.627
TA11	92	685	565	0.41	−0.083	−0.742

## Data Availability

The original contributions presented in the study are included in the article, further inquiries can be directed to the corresponding authors.

## Appendix A. Hyperparameter Settings for Machine Learning Models

To ensure the reproducibility of the results presented in this study, Table A1 summarizes the hyperparameter settings used for all machine learning models.

**Table A1:** Hyperparameters for machine learning models.

Model	Parameter	Value
GA-HL-XGBoost	Learning rate (*η*)	0.1
	Maximum tree depth	6
	Subsample ratio	0.8
	Column sampling ratio	0.8
	Regularization parameter (*λ*)	1
	Loss function	Pseudo-Huber loss
	Population size	50
	Number of iterations	100
	Crossover rate	0.9
	Mutation rate	1/Lind
	Selection strategy	Ranking fitness, tournament
GA-RF	Population size	50
	Number of iterations	100
	Crossover rate	0.9
	Mutation rate	1/Lind
	Selection strategy	Tournament selection
	Number of trees	100
	Maximum depth	12
Deep Random Forest	Number of layers	4
	Trees per layer	200
	Maximum depth per tree	12
	Early stopping criteria	Accuracy improvement < 0.5%
Blending Model	Model components	GA-HL-XGBoost, GA-RF, DRF
	GA-HL-XGBoost weight	0.45
	GA-RF weight	0.35
	DRF weight	0.20

## Appendix B. Hyperparameter Settings for DDPG Algorithm

Table A2 provides the detailed hyperparameter settings used for the deep deterministic policy gradient (DDPG) algorithm applied to solve the improved SWT equation.

**Table A2:** Hyperparameters for the DDPG algorithm.

Component	Parameter	Value
Policy Network	Hidden layers	[128, 64]
	Activation function	ReLU
	Learning rate	0.001
Value Network	Hidden layers	[128, 64]
	Activation function	ReLU
	Learning rate	0.002
Training Parameters	Batch size	64
	Discount factor (*γ*)	0.99
	Soft update rate (*τ*)	0.001
	Replay buffer size	100,000
Exploration Noise	Noise type	Ornstein Uhlenbeck
	Mean	0
	Standard deviation	0.2
Reward Function	Error weight (*α*)	1.0
	Deviation weight (*β*)	0.5
	Iteration cost weight (*γ*)	0.01
Convergence Criteria	Maximum iterations	200
	Tolerance	10^−6^
